# Quantifying crowd size with mobile phone and *Twitter* data

**DOI:** 10.1098/rsos.150162

**Published:** 2015-05-27

**Authors:** Federico Botta, Helen Susannah Moat, Tobias Preis

**Affiliations:** 1 Centre for Complexity Science, Warwick Business School, University of Warwick, Coventry CV4 7AL, UK; 2 Data Science Lab, Behavioural Science, Warwick Business School, University of Warwick, Coventry CV4 7AL, UK

**Keywords:** data science, computational social science, complex systems

## Abstract

Being able to infer the number of people in a specific area is of extreme importance for the avoidance of crowd disasters and to facilitate emergency evacuations. Here, using a football stadium and an airport as case studies, we present evidence of a strong relationship between the number of people in restricted areas and activity recorded by mobile phone providers and the online service *Twitter*. Our findings suggest that data generated through our interactions with mobile phone networks and the Internet may allow us to gain valuable measurements of the current state of society.

## 2. Introduction

The ability to quickly and accurately estimate the size of a crowd is crucial in facilitating emergency evacuations and avoiding crowd disasters [1]. However, existing approaches which rely on human analysts counting samples of the crowd can be time-consuming or costly [2]. Similarly, image-processing solutions require image data to be available in which members of the crowd can be identified and counted by an algorithm [3,4]. Recent studies have provided evidence that data generated through interactions with the Internet [5–20], mobile phone networks [21–25] and other large technological systems can offer new insights into human behaviour [26–31]. Here, we investigate whether data on mobile phone usage and usage of the online social media service *Twitter* can be used to estimate the number of people in a specific area at a given time. We consider data resulting from ordinary use of smartphones, without the need for users to install specific applications on their mobile phone.

## 3. Data

**Figure 1.**
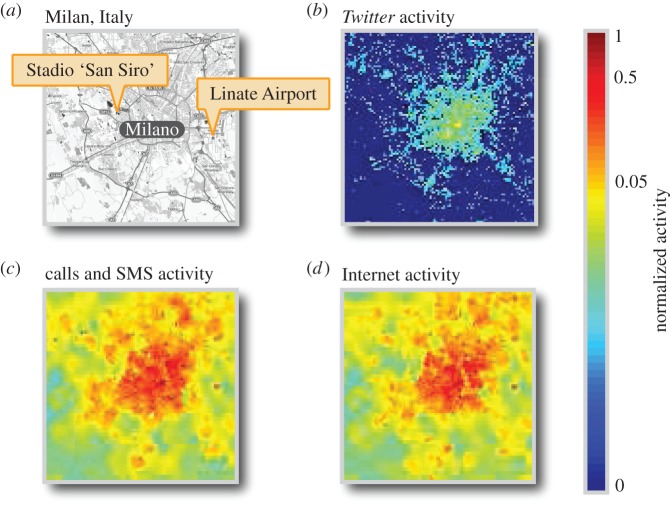
*Twitter*, calls and SMS, and Internet activity in Milan. (*a*) We analyse *Twitter*, calls and SMS, and Internet activity data recorded from mobile phones in the city of Milan and surroundings. The geographical area around Milan for which all these datasets are available is represented in this map, created using data from OpenStreetMap. The datasets cover the period from 1 November 2013 to 31 December 2013. We aim to determine whether such mobile phone data can be used to infer the number of people in a specific location at a specific time. To calibrate our model, we consider two case studies: San Siro football stadium and Linate Airport. (*b*) We depict the normalized number of tweets recorded during the first week of November 2013, for the geographical area shown in (*a*). Tweet counts are extracted from the full set of geolocalized tweets sent during this period. We observe a higher density of tweets in the centre of Milan. (*c*) We depict normalized data on the total number of calls made and received as well as text messages (SMS) sent and received during the time interval between 08.20 and 08.30 of 1 November 2013, for the geographical area depicted in (*a*). We again observe more activity in the centre of Milan. (*d*) We depict normalized data on the number of requests made by mobile phones to access the Internet during the time interval between 08.20 and 08.30 of 1 November 2013, for the geographical area shown in (*a*). Visual inspection of this dataset provides further evidence that more mobile phone activity is recorded in locations where greater numbers of people would be expected. Colours in (*b*–*d*) are normalized to the maximum recorded activity level in each dataset.

We retrieve data on mobile phone and *Twitter* activity recorded in the city of Milan and surroundings in a period covering two months from 1 November 2013 to 31 December 2013 [32]. Both datasets describe activity in the geographical area depicted in figure 1*a*. The *Twitter* dataset consists of all messages sent via *Twitter* (‘tweets’), with associated geographical coordinates located within the area shown in figure 1*a*. Tweets are also timestamped. Initial visual inspection of the *Twitter* data shows that greater numbers of tweets are recorded in the centre of Milan, where we would expect greater numbers of people to be found (figure 1*b*).

The mobile phone activity dataset describes the volume of calls made and received, SMSs sent and received and Internet connections opened, closed and maintained. Mobile phone activity measurements are provided at 10 min granularity, for cells in a discrete grid superimposed on the area of Milan. This grid has 10 000 cells of size 235×235 . Further details of this dataset are provided in the electronic supplementary material. Visual inspection of the distribution of call and SMS activity (figure 1*c*) and Internet connection activity (figure 1*d*) again confirms mobile phone activity is highest in the city centre of Milan.

## 4. Results

We investigate whether the information present in these datasets can be used to infer the number of people in specific areas of Milan at a given time. To calibrate our model, we consider two case studies of access restricted areas for which relevant data exist: San Siro football stadium, for which we have attendance counts for 10 football matches which took place during the period of analysis, and Linate Airport, for which we use flight schedule data to create a proxy indicator for the number of people present in the airport at any given time.

**Figure 2.**
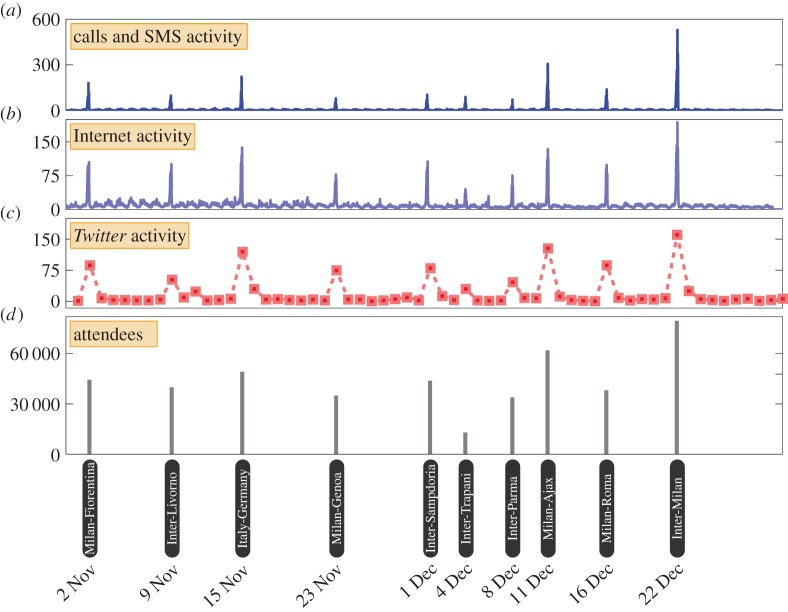
Mobile phone and *Twitter* activity in football stadium Stadio San Siro. (*a*) We depict the time series of mobile phone call and SMS activity recorded in the cell in which the football stadium is located, during the period of analysis between 1 November 2013 and 31 December 2013. The time series is plotted at 10 min granularity. (*b*) Similarly, we depict the time series of Internet connection activity in the cell in which Stadio San Siro is located, at 10 min granularity. (*c*) Finally, we depict the daily counts of tweets recorded within the vicinity of the stadium. (*d*) We determine the dates of football matches which took place during this period, and plot the number of attendees which were recorded at each of these matches. Visual inspection reveals a remarkable alignment between the spikes that can be observed in the communication activities and the dates on which football matches took place. The heights of the spikes bear a strong similarity to the number of attendees at each match.

We examine the time series of call and SMS activity (figure 2*a*), Internet activity (figure 2*b*) and *Twitter* activity (figure 2*c*) recorded in the vicinity of the football stadium Stadio San Siro during the period of analysis between 1 November 2013 and 31 December 2013. The coordinates of the area for which data were analysed are given in the electronic supplementary material, tables S3 and S4. In all three time series, we observe 10 distinct spikes, which occur at the same times across all time series. We find that the dates on which these spikes occur coincide exactly with the dates on which the 10 football matches took place in the stadium during this period (figure 2*d*). Furthermore, we note that the relative sizes of the spikes in the mobile phone and *Twitter* activity time series (figure 2*a*–*c*) bear a strong similarity to the relative sizes of the attendance counts for these matches, as depicted in figure 2*d*.

**Figure 3.**
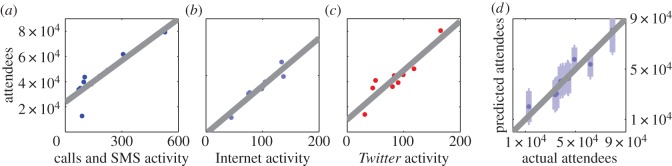
Comparing football match attendance figures to mobile phone and *Twitter* activity. (*a*) We investigate whether there is a relationship between the number of people attending each football match and the recorded mobile phone call and SMS activity inside the stadium. We find a linear relationship between these two variables (adjusted *R*^2^=0.771, *N*=10, *p*<0.001, ordinary least-squares regression). (*b*) Similarly, we find a pattern consistent with a linear relationship between Internet connection activity in the stadium and the number of attendees at each match (adjusted *R*^2^=0.937, *N*=10, *p*<0.001, ordinary least-squares regression). (*c*) We also observe a linear relationship between *Twitter* activity in the stadium and the number of match attendees (adjusted *R*^2^=0.855, *N*=10, *p*<0.001, ordinary least-squares regression). (*d*) We explore whether this relationship could be exploited to infer the number of attendees from communication data if no other measurements were available. Using data on Internet activity, we build a linear regression model using only nine out of the 10 football matches and then predict the attendance at the 10th match. We then repeat this leaving a different match out every time. Here, we plot the resulting estimates and their 95% prediction intervals. We find that the actual number of attendees falls within the 95% prediction interval for all 10 matches.

We extract the maximum values of the spikes in calls and SMS activity, Internet activity and *Twitter* activity. We observe a linear relationship between the number of people attending the football matches and the volume of incoming and outgoing phone calls and SMS messages (adjusted *R*^2^=0.771, *N*=10, *p*<0.001, ordinary least-squares regression; figure 3*a*). We find similar relationships between the number of attendees and both Internet activity (*R*^2^=0.937, *N*=10, *p*<0.001, ordinary least-squares regression; figure 3*b*) and *Twitter* activity (*R*^2^=0.855, *N*=10, *p*<0.001, ordinary least-squares regression; figure 3*c*). While figure 3*a*–*c* suggest a strongly linear relationship between mobile phone activity data and the number of attendees, we note that this relationship holds in a non-parametric analysis too (calls and SMS activity: Spearman's *ρ*=0.927, *N*=10, *p*<0.001; Internet activity: Spearman's *ρ*=0.976, *N*=10, *p*<0.001; *Twitter* activity: Spearman's *ρ*=0.924, *N*=10, *p*<0.001).

We investigate the possibility of using the information present in communication data to infer the number of attendees in situations where no other measurements are easily accessible. As an example, we consider data on Internet activity, for which the relationship with the number of recorded attendees was strongest. We carry out a *leave-one-out cross-validation* analysis as follows: for each of the 10 attendance figures, we build a linear regression model based on the remaining nine attendance figures and the corresponding Internet activity data. We then use this model to generate an estimate of the attendance figure which was removed from the recorded Internet activity data. In figure 3*d*, we plot the resulting estimates and their 95% prediction intervals. We find that the actual attendance figure is always within the 95% prediction interval of our estimate.

We note that our analysis of mobile phone activity data may be affected by capacity constraints, such as signal truncation, on mobile phone communication in the stadium. Should data on such constraints become available in the future, the influence of these constraints on the relationship between communication data and crowd size may merit further analysis.

We perform a parallel analysis of the relationship between mobile phone and *Twitter* data and the number of passengers at Linate Airport. To estimate the number of people in Linate Airport at any given hour during the analysis period, we assume that passengers may arrive at the airport up to 2 h before a departing flight, and depart within an hour following a flight arrival. For each hour, we therefore calculate the number of flights departing in the following 2 h or arriving in the previous hour, and use this as a proxy indicator for the number of passengers in the airport. We base our calculations on one week of flight schedule data from May 2014, as explained in the electronic supplementary material, and assume that weekly flight schedules are relatively constant. Our proxy indicator is therefore calculated for each of the 168 hours in a week. We omit the three initial days and two final days of the analysis period to create a period of exactly eight weeks.

**Figure 4.**
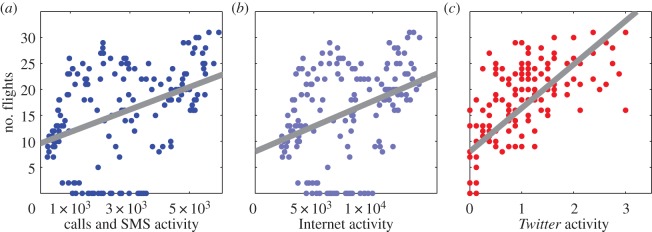
Parallel analysis of the relationship between mobile phone and *Twitter* data and the number of passengers at Linate Airport. (*a*) We create a proxy indicator for the number of passengers at Linate Airport in each hour by calculating the number of flights departing in the following 2 h or arriving in the previous hour. We compare this proxy indicator to the average mobile phone call and SMS activity recorded for each hour in a week, in the cells in which the airport is located. We find that greater activity corresponds to a greater estimated number of passengers (adjusted *R*^2^=0.175, *N*=168, *p*<0.001, ordinary least-squares regression). The relationship we find is weaker than that found for the football attendance figures, but remarkable given the coarse nature of our estimate of the number of passengers. (*b*) We then explore the relationship between the proxy indicator of the number of passengers and Internet connection activity recorded in the cells in which the airport is located. Again, we find that greater Internet activity corresponds to a higher number of passengers (adjusted *R*^2^=0.143, *N*=168, *p*<0.001, ordinary least-squares regression). (*c*) As a final example, we consider *Twitter* activity recorded in the cells in which the airport is located. Again, we consider the average number of tweets recorded during each of the 168 hours in a week, over the eight week period of our analysis. Here, we find a stronger relationship between the estimated number of passengers and activity on *Twitter* (adjusted *R*^2^=0.510, *N*=168, *p*<0.001, ordinary least-squares regression).

We compare this proxy indicator to the average mobile phone call and SMS activity and to the average Internet activity recorded for each hour in a week, in the cells in which the airport is located, as detailed in the electronic supplementary material, table S5. We find that greater phone call and SMS activity corresponds to a greater estimated number of passengers (adjusted *R*^2^=0.175, *N*=168, *p*<0.001, ordinary least-squares regression; figure 4*a*). Similarly, we find that greater Internet activity relates to a higher estimated number of passengers (adjusted *R*^2^=0.143, *N*=168, *p*<0.001, ordinary least-squares regression; figure 4*b*). The relationships we find are weaker than those found in the previous case study, but remarkable given the coarse nature of our estimate of the number of passengers.

We analyse *Twitter* activity in the area of the airport detailed in the electronic supplementary material, table S6. In this case, we observe a stronger relationship between the estimated number of passengers and activity on *Twitter* (adjusted *R*^2^=0.510, *N*=168, *p*<0.001, ordinary least-squares regression; figure 4*c*). We observe that mobile phone, SMS and Internet activity is still recorded when no flights take place, generally during night-time periods. By contrast, few tweets are logged at these times, potentially explaining the greater strength of this relationship.

We note that roughly 58% of the passengers travelling to and from Linate Airport are Italian [33]. Given the current costs of using mobile phone networks abroad, the mobile phone activity analysed here may reflect the behaviour of Italian passengers more strongly than the behaviour of international passengers.

## 5. Conclusion

Our results provide evidence that accurate estimates of the number of people in a given location at a given time can be extrapolated from mobile phone or *Twitter* data, without requiring users to install further applications on their smartphones. As well as being of clear practical value for a range of business and policy stakeholders, our findings suggest that data generated through our interactions with mobile phone networks and the Internet may allow us to gain valuable measurements of the current state of society.

## Supplementary Material

Supplementary material with additional information on the data sets.

## Data accessibility

Datasets used in this study are available via the Dryad Repository (doi:10.5061/dryad.1rk60).
