# Supplementary material for: Quantifying crowd size with mobile phone and Twitter data
Source: R Soc Open Sci. 2015 May 27;2(5):150162. doi: 10.1098/rsos.150162 (PMC4453255; doi:10.1098/rsos.150162)
Supplement: Supplementary material with additional information on the data sets. [file rsos150162supp1.pdf]

Quantifying Crowd Size with Mobile Phone and *Twitter* Data
Supplementary Material

Federico Botta,[1,2*] Helen Susannah Moat,[2] Tobias Preis[2]

[1] Centre for Complexity Science, University of Warwick, Coventry, CV4 7AL, UK

[2] Data Science Lab, Behavioural Science, Warwick Business School,
University of Warwick, Coventry, CV4 7AL, UK

*To whom correspondence should be addressed; E-mail: f.botta@warwick.ac.uk.

Mobile phone data We retrieved data on mobile phone call, SMS and Internet activity in Milan and surroundings from 1 November 2013 until 31 December 2013 from `http://www.telecomitalia.com/bigdatachallenge` as part of the *Big Data Challenge* set up by *Telecom Italia*.

Interactions with the *Telecom Italia* mobile network generate Call Detail Records (CDRs). In the dataset we consider, *Telecom Italia* provides data on CDRs relating to the following activities:

- SMS: a CDR is generated for every SMS which is sent and every SMS which is received

- Calls: every incoming and outgoing call generates a CDR
- Internet access: a CDR is generated for each of the following events:
  - An Internet connection is opened
  - An Internet connection is closed
  - An Internet connection is open and 15 minutes has passed since the last CDR
  - An Internet connection is open and 5 MB have been transferred since the last CDR

For privacy reasons, the values which *Telecom Italia* provides are rescaled using an unknown factor. *Telecom Italia* specifies that counts of mobile phone call and SMS CDRs are rescaled using the same factor, and are therefore comparable. Counts of Internet activity CDRs are rescaled using a different factor.

Similarly, we retrieved the complete set of geo-localised tweets posted in Milan and surroundings between 1 November 2013 and 31 December 2013 from `http://www.telecomitalia.com/bigdatachallenge`.

Football match attendees We retrieved football match attendance figures from the following websites:

- Seven of the ten games which took place during the period of analysis were part of the Italian National Football League 'Serie A'. We retrieved attendance figures from the official website of the 'Serie A': `http://www.legaseriea.it/it/lega-calcio/regolamenti-e-documenti/dati-statistici-su-incassi-e-spettatori`

- Attendance figures for the three remaining games that took place during this period were retrieved from the following URLs of two online newspapers:

  - `http://www.calciomercato.com/news/inter-trapani-3-2-il-tabellino-919259`
  - `http://www.milannews.it/il-match/quasi-cinquantamila-spettatori-riempiono-san-siro-105483`
  - `http://www.milannews.it/il-match/milan-ajax-superati-i-61mila-spettatori-a-san-siro-130840`

Airport data Flight schedule data for *Linate Airport* can be retrieved from `http://www.milanolinate-airport.com/it` for the current date and the following four days. In May 2014, we retrieved the flight schedule for the week between Monday 5 May 2014 and Sunday 11 May 2014. We assume that weekly flight schedules are reasonably constant across time, and use the schedule retrieved for this week as a proxy for the flight schedule in the weeks between 1 November 2013 and 31 December 2013.

Table S1. Full names of football teams. The full names of the football teams referred to in our analysis.

**Abbreviation**	**Full Name**
Milan	A.C. Milan
Inter	F.C. Internazionale Milano
Fiorentina	A.C.F. Fiorentina
Livorno	A.S. Livorno Calcio
Italy	Italy National Football Team
Germany	Germany National Football Team
Genoa	Genoa C.F.C.
Sampdoria	U.C. Sampdoria
Trapani	Trapani Calcio
Parma	Parma F.C.
Ajax	AFC Ajax
Roma	A.S. Roma

Table S2. *San Siro* football match attendance figures. Attendance figures for the football matches analysed.

Match	Attendees
Milan-Fiorentina	44261
Inter-Livorno	39775
Italy-Germany	49000
Milan-Genoa	34848
Inter-Sampdoria	43607
Inter-Trapani	12714
Inter-Parma	33732
Milan-Ajax	61744
Milan-Roma	37987
Inter-Milan	79311

Table S3. Coordinates of the area around *San Siro* for which data on phone calls, SMS and Internet activity was retrieved. This corresponds to one cell in the *Telecom Italia* dataset. Coordinates are specified using the WGS84 coordinate system. Note that the *Telecom Italia* cells do not appear precisely square using this system.

Corner	Latitude in WGS84	Longitude in WGS84
Top left	45.4793078474071	9.12276821006816
Top right	45.479304576233446	9.125775032395008
Bottom right	45.477189306362206	9.125770326481447
Bottom left	45.477192577295924	9.122763616655133

Table S4. Coordinates of the area around *San Siro* for which *Twitter* data was retrieved. This area constitutes a bounding box around the *San Siro* stadium, including the entrance gates.

Corner	Latitude in WGS84	Longitude in WGS84
Top left	45.480084	9.121097
Top right	45.480084	9.125807
Bottom right	45.476308	9.125807
Bottom left	45.476308	9.121097

Table S5. Coordinates of the area around *Linate Airport* for which data on phone calls, SMS and Internet activity was retrieved. This corresponds to a square of nine cells in the *Telecom Italia* dataset, centered around the airport.

Corner	Latitude in WGS84	Longitude in WGS84
Top left	45.464233335498925	9.27604292987004
Top right	45.464211186534364	9.285060903904881
Bottom right	45.45786542106381	9.285028927452782
Bottom left	45.45788756515503	9.276011964969305

Table S6. Coordinates of the area around *Linate Airport* for which *Twitter* data was retrieved. This area corresponds to the square of nine cells around the airport, but corner coordinates are modified slightly to produce a square area under the WGS84 coordinate system.

**Corner**	**Latitude in WGS84**	**Longitude in WGS84**
Top left	45.464233335498925	9.276011964969305
Top right	45.464233335498925	9.285060903904881
Bottom right	45.45786542106381	9.285060903904881
Bottom left	45.45786542106381	9.276011964969305
